# *De novo-*assembled long-read genomes of *Saccharomyces cerevisiae* strains in commerce

**DOI:** 10.1128/mra.00705-24

**Published:** 2024-12-12

**Authors:** P. S. Shwed, V. Anoop, G. Leveque

**Affiliations:** 1 Environmental and Radiation Health Sciences Directorate, Healthy Environments and Consumer Safety Branch, Health Canada, Ottawa, Ontario, Canada; 2 New Substances Assessment and Control Bureau, Safe Environments Directorate, Healthy Environments and Consumer Safety Branch, Health Canada, Ottawa, Ontario, Canada; 3 Canadian Centre for Computational Genomics (C3G), McGill University, Montréal, Quebec, Canada; 4 Department of Human Genetics, McGill University, Montreal, Quebec, Canada; University of California Riverside, Riverside, California, USA

**Keywords:** baking yeast, wine-making yeast, genomes

## Abstract

*Saccharomyces cerevisiae* strains NRRL Y-1646, Red Star, and VL3 are commercial yeast strains for baking and fermentation. These long-read genome assemblies will serve to better understand structural variation and biological properties of these strains.

## ANNOUNCEMENT

*Saccharomyces cerevisiae* is a yeast species that is found in diverse indoor and outdoor environments and used in food and beverage fermentation since ancient times (1) and more recently in the production of enzymes and biochemicals (2). The complete long-read genome sequences of two baking (NRRL Y-1646 and Red Star) strains and one wine-making (VL3) strain were collected as part of research to support the risk assessment of products of biotechnology in Canada. Long-read sequences were collected in order to resolve genome structural variations. NRRL Y-1646 (Garey F53; F53) strain was provided by the USDA-ARS Culture Collection (NRRL). Red Star (RS) active dry yeast (Lesaffre, USA) and VL3 (Laffort, USA) strains were purchased. All strains were grown on YPD agar (per liter: 10 g yeast extract, 20 g peptone, 20 g dextrose, and 15 g Bacto agar). Single colonies were inoculated in 2 mL yeast extract–peptone–dextrose (YPD) liquid broth and cultured for 24 hours at 30°C. Genomic DNA was extracted using the MasterPure Yeast DNA Purification Kit (Epicentre, USA).

Large insert libraries (>10 kb) for strains F53, RS, and VL3 were made by shearing genomic DNA using Covaris g-TUBES (Covaris Inc., USA).  For strains F53 and VL3, the sheared genomic DNA was purified using AMPure PB magnetic beads (Pacific Biosciences (PacBio), USA), followed by size selection on a BluePippin system (Sage Science Inc., USA) using a cutoff range of 15 kb to 50 kb. Libraries were constructed using SMRTbell express template prep kit v1.0 (PacBio, CA) according to the manufacturer’s protocol.

Genome sequences for strains F53 and VL3 were collected on a PacBio RSII system instrument (PacBio, USA) with single-molecule real-time (SMRT) Cells v3 using a 4 hour movie collection time. *De novo* assembly, read error correction, and trimming of reads were carried out with the Canu assembler v1.3 using default settings (3).

For strain VL3, the DNA library was generated using SMRTbell Express Template Prep Kit 2.0 (PacBio, USA) following the manufacturer’s standard protocol. Genomic libraries were size-selected for >10 kb using the Blue Pippin system (Sage Science Inc., USA), loaded on an SMRT cell 8M tray, and sequenced on a Sequel II system instrument using a 10 hour movie collection time with Sequel II Sequencing Kit 2.0. *De novo* assembly of raw uncorrected reads was carried out with the Flye assembler (v. 2.9.4) using default settings (4), and Quast v.5.2.0 was used for GC content and fragment size estimation (5) .

The completeness of each assembly was judged via BUSCO v5.2.2 analysis using default settings and the “saccharomycetes_odb10” data set that contained 2137 benchmarking universal single-copy orthologs (BUSCOs) (6)

Genome assembly statistics are shown in Table 1. These genomes will serve as a resource for understanding genome variations and biological properties of commercial *S. cerevisiae* strains.

**TABLE 1:** Genome assembly metrics and accession numbers

Feature	Garey F53	Red Star	VL3
GenBank accession no.	JAKZJO000000000	JAKZJP000000000	JAKZJN000000000
SRA accession no.	SRR18050280	SRR18050281	SRR18050279
SRA raw read number	128,727	356,268	5,564,511
Assembled genome size (Mb)	12.8	12.3	12.3
Fold-coverage (total bp/genome size)	65.0x	233.0x	4,344x
Assembly N50 (kb)	548.3	441.7	802.2
Number of contigs	53	62	31
G+C content (%)	38	38.5	37.5
Complete and single-copy BUSCOs (%)	85.3	96.5	96.1

## Data Availability

The whole genome sequences were deposited in DDBJ/ENA/GenBank under the BioProject PRJNA704095, and the accessions are listed in Table 1. The raw sequencing reads were deposited in the NCBI SRA associated with BioProject PRJNA704095 and are listed in Table 1.
